# Supplementary material for: Effects of chemotherapy on skeletal muscle mitochondrial oxidative capacity using near-infrared spectroscopy (NIRS): Protocol paper for an observational mixed model repeated measures design in patients with breast and gynecological cancer
Source: PLoS One. 2025 Jun 27;20(6):e0315351. doi: 10.1371/journal.pone.0315351 (PMC12204469; doi:10.1371/journal.pone.0315351)

Near Infrared Spectroscopy (NIRS) as a Method for Measuring Oxidative Capacity of Skeletal Muscle Mitochondria in Breast Cancer and all Gynecological Cancer Patients Protocol

6/24/22

Participants. Participants recruited for this study will be breast cancer or gynecological cancer patients (N=40) who agree to participate in an exercise study examining the effects of chemotherapy on muscle mitochondrial oxidative capacity, a measure of skeletal muscle health. A written informed consent will be obtained from the participants before data collection. This study has been submitted for review by the Institutional Review Board at the Prisma Office of Research Compliance and Administration (ORCA). We pledge to abide by all applicable institutional and governmental regulations regarding the ethical use of human volunteers for the duration of this research.

This study focuses on breast cancer or gynecological cancer patients who will undergo a standard of care chemotherapy treatment as directed by their oncologist.

Other eligibility requirements for this study are as follows:

- Patients diagnosed with breast cancer or gynecological cancer without distance metastasis
- Ages > 20 years old
- Able to perform exercise on a stationary cycle ergometer at moderate intensities for a maximum of 15 minutes
- Hemoglobin values greater than 10 at baseline
- ALT and AST values less than 2.5X the upper limit of normal by institutional standards
- Godin-Shepard Leisuretime Physical Activity Questionnaire (GLTEQ) score of 14 or greater will be included. A score of less than 14 is considered insufficiency active/sedentary.

The exclusion criteria include patients with:

- Clinically advanced cardiovascular disease
- Clinically advanced pulmonary disease
- Disease requiring continuous oxygen supplementation
- Greater than 2 centimeters or more of subcutaneous adipose tissue on the anterior thigh
- Inability to walk or stand
- Movement disorders
- Spinal cord injuries
- Autoimmune disorders
- Pregnant or breastfeeding
- Mini-Mental State Examination (MMSE)

Participants for this study will be recruited through informative pamphlets at the Center for Integrative Oncology Survivorship, Prisma Health System’s Cancer Institute at Greenville Memorial Hospital and affiliated physician offices.

Experimental Design. We will use an observational, 2-way, treatment x time, within-subjects research design to compare skeletal muscle oxidative capacity of the vastus lateralis muscle, during stationary cycling, across breast cancer or gynecological cancer patients at baseline and across their chemotherapy regimen. Muscle mitochondrial oxidative capacity of the Vastus

Lateralis muscle will be non-invasively evaluated using continuous wave, Near Infrared Resonance Spectroscopy (NIRS, PortaMon by Artinis Medical Systems BV in The Netherlands).

Study protocol. The NIRS participant testing will be performed in the University of South Carolina School of Medicine Greenville's Human Performance Laboratory located within Cancer Institute Faris Road facility. Testing will take approximately 30 minutes per session, with a maximum of 180 minutes of participation per patient. The independent variables in this study are drug regimen and number of chemotherapy treatments. The dependent variables in this study will be the change in oxygenation and deoxygenation status of the vastus lateralis as measured by the NIRS device, indicative of mitochondrial oxidative capacity, after a bout of cycling. Patients will be asked to avoid exercise and use of certain substances (tobacco, alcohol, caffeine, and contraindicated medications) 24 hours prior to testing.

The NIRS device consists of three LED transmitters that transmit light waves into muscle tissue and one high sensitivity PIN diode receiver, equipped with ambient light protection, which assesses light received from the transmitters. The three transmitters each emit light at two wavelengths. These wavelengths correspond to the absorption wavelengths of oxyhemoglobin (O_2Hb) and deoxyhemoglobin in the skeletal muscle tissue (approximately 760 nm and 850 nm). These wavelengths of light will be received from three separate transmitter distances (30mm, 35mm, and 40mm) at 10 Hz. The ΔO_2Hb and Δ tissue saturation index (TSI%) will be calculated as the change from the last 30-s average of the work interval to the last 30-s average of the recovery interval. Oxygen uptake (VO_2) will be obtained by using open-circuit spirometry (Parvo Medics, Inc, Salt Lake City, Utah). Cycling power will be determined via lactate threshold curves generated for each participant.

Participants will be asked to perform exercise in shorts or equivalent. Participants will first sit at rest in a chair, as the NIRS device is applied to the vastus lateralis. The device will have double sided adhesive tape to lightly secure its position to the skin. The device will be wrapped in a black cloth to fully secure its position, limit device mobility, and block ambient light in the laboratory. Participants will then stand and walk to the cycle with the NIRS secured to the participant's leg. Participants will sit on the bike for a 2-minute rest while wearing a fitted mask that comfortably measures the participant's rate and amount of oxygen uptake (VO_2) and other ventilatory measurements at rest and during exercise.

Visit #1. Researchers will determine the participant's lactate threshold curve and ventilatory threshold during a graded exercise test (Table 1). The lactate threshold is a measure of the rate at which capillary blood lactate measurements rise as an effect of increased exercise intensity. The blood lactate curve will be used to determine an individualized power wattage for subsequent exercise testing of "on-off kinetics" (see below description). Capillary blood lactate levels will be obtained by finger stick for point of care testing. Ventilatory threshold will be measured using indirect calorimetry via a metabolic cart that captures expired gasses (O_2 and CO_2) through a comfortable silicone mask worn by the participant.

A baseline blood lactate level will be obtained at rest, then the participant will be instructed to cycle for three minutes beginning at 10W. After the 3-minute stage is complete, the power

output will increase by 15 watts. The participant will cycle for 3 minutes at each stage until their lactate threshold reaches 4.0 mmol/L. Once the blood lactate reaches 4.0 mmol/L, one final, three-minute stage will be performed. A final lactate reading will be obtained at the completion of this exercise stage. The power output that is equivalent to 1.1x the wattage at which their lactate threshold exceeded 4.0 mmol/L will be used as the exercise intensity (“on-kinetics”) for subsequent visits accompanying chemotherapy treatments.

Subsequent Visits. Participants will return for each subsequent visit within a week before their next chemotherapy infusion as long as they are able to safely continue the exercise protocol (Table 2). The NIRS device will be secured to the participant’s leg similar to the first visit and the participants will put on the mask to measure ventilation. Participants will begin pedaling for a warmup at 0 watts for 2 minutes. After the two-minute warm up period is completed, the power output will increase to the previously calculated wattage of 1.1x LT. The participant will cycle at a self-selected cadence at this power wattage for a duration of 2 minutes (“on-kinetics”). At the end of each on-kinetics stage, participants will be asked to rate their level of effort during exercise, Rate of Perceived Exertion (RPE). The participant will then stop pedaling and will remain in recovery, sitting on the bike for 2 minutes (“off-kinetics” stage). With 5 seconds remaining in the “off-kinetics” stage, the participant will start pedaling at the same self-selected cadence for their next “on-kinetics” stage, followed by another 2-min “off-kinetics” period. This cycle of exercise followed by rest, known as on-off kinetics, will repeat for a total maximum of 3 times, or until the participant reaches volitional fatigue.

Collection of surveys and biometrics at each visit. We will be administering MoCA, BFI, Physical Activity Intake, PROMIS Global Health, and GLTPAQ surveys prior to participant engagement in cycling exercises. We will also be administering BFI, PROMIS Global Health, and Physical Activity Follow-up surveys after each individual study session to assess changes in cancer related fatigue associated with chemotherapy treatment that may develop throughout the study time period.

Patients will undergo routine, standard of care blood draws for a CBC and metabolic testing per their managing physician with an additional tube, during the same venipuncture, for a study covered aliquot for biomarkers related to mitochondrial health for correlative research.

For biomarker analysis, exosomes from patient serum (approximately 250 uL samples) will be isolated using an ExoQuick ULTRA EV Isolation System (System Biosciences). Exosomes will then be detected with Western blotting with exosome specific antibodies. Exosome samples will be sent for RNASeq analysis of RNA within the isolated exosomes at System Biosciences. Bioinformatics analysis of the RNAs identified from the RNASeq will then be used to determine if there are specific RNAs present in patient blood associated with changes in mitochondrial function. In addition, selected mRNAs associated with mitochondrial function will be assessed (expression levels determined) with RT-qPCR using aliquots of the isolated serum exosomes.

Statistical Analysis. ANOVA one way with tukey post-hoc analysis to detect differences between drug regimen groups at each time point (baseline, all subsequent chemotherapy treatments) for time constant response and change in mitochondrial function for mitochondrial oxidative capacity. One-way ANOVAs will be used to additionally detect differences between different timepoints from baseline to final chemotherapy treatment for each individual chemotherapy regimen.

Table 1. Lactate Threshold and Power Performance

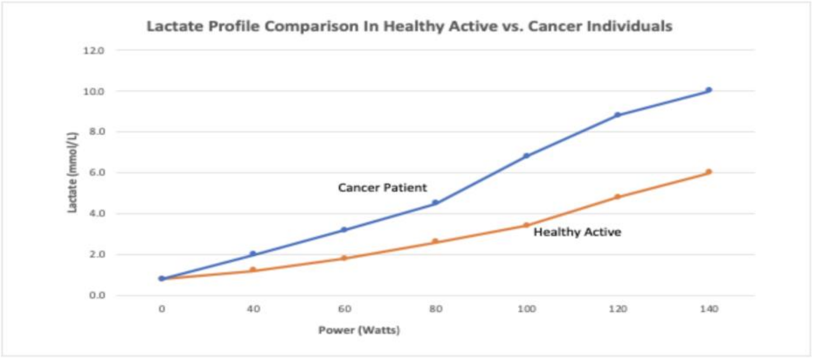

Brian Leary, 2020.

Table 2.

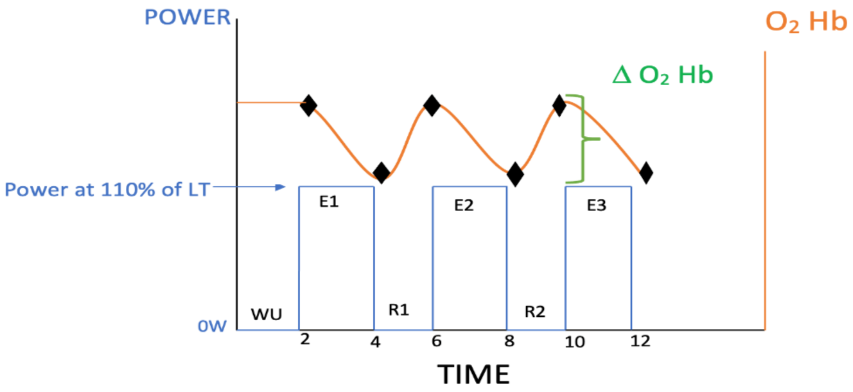

Supplement: S1 File — (PDF) [file pone.0315351.s002.pdf]
